# Knowledge of Thyroid Disease Manifestations and Risk Factors Among Residents of the Eastern Province, Saudi Arabia

**DOI:** 10.7759/cureus.13035

**Published:** 2021-01-31

**Authors:** Abdulwahab Alyahya, Abdulrahman AlNaim, Abdulelah W AlBahr, Fawaz Almansour, Ahmed Elshebiny

**Affiliations:** 1 Internal Medicine Department, College of Medicine, King Faisal University, Al Ahsa, SAU; 2 Internal Medicine, Diabetes and Endocrinology, Faculty of Medicine, Menoufia University, Shebin El Kom, EGY; 3 Internal Medicine Department, Diabetes and Endocrinology, King Faisal University, Al Ahsa, SAU

**Keywords:** thyroid disorder, risk factors, knowledge, eastern province, saudi arabia

## Abstract

Background: Thyroid disorders are worldwide common endocrine disorders. These disorders are frequently under-diagnosed. In general, lack of knowledge and understanding of thyroid disorder effects can lead patients to go undiagnosed. This study aims to figure out the level of the general knowledge about thyroid disease manifestations, risk factors, and preventive behaviors among the general population.

Method: This cross-sectional study was conducted among 882 participants in the Eastern province of Saudi Arabia from July 2020 to October 2020, and were selected randomly. The representative sample included Saudi and non-Saudi males and females of different age groups (18-60 years).

Result: The overall mean knowledge score was 8.67 (SD 3.69) with 44.7%, 41.2%, and 14.2% were classified into low, average, and high knowledge, respectively. In the comparison of mean knowledge score among the socio-demographic profiles and previous history of thyroid disease, the current study shows that being a female, living in Al Ahsa, being a student, those with a previous history of thyroid disease, family history of the disease, and those who underwent thyroid gland examination were significantly more associated with having better knowledge toward thyroid diseases.

Conclusion: Nearly, half of the studied sample has low awareness scores regarding thyroid disease manifestations and its risk factors. The health authorities should hold more successful health education methods to improve the public and their caregivers' awareness of the various aspects of thyroid disorders and the value of their early detection and adequate control.

## Introduction

The thyroid gland is considered the largest endocrinal gland in the human body, located in the anterior aspect of the neck. It synthesizes and releases thyroid hormones that considerably influence the basal metabolic rate (BMR) and protein synthesis [1]. Furthermore, these hormones are also critical for children and adolescents' neurocognitive development and maintaining normal physiological functioning in adults [2].

Thyroid disorders are conditions that result from either over/under-secretion of thyroid hormones as well as thyroid gland enlargement. Thyroid disorders can be primary (directly related to the gland itself) or secondary (thyroid dysfunction due to other factors). These disorders were reported in more than 110 countries, with 1.6 billion people at risk [3]. Thyroid disorders are one of the most common medical conditions worldwide [4]. Iodine deficiency is a major cause of thyroid disorders. It is estimated that about one-third of the world population lives in an iodine-deficient area, and over 190 million suffer from iodine deficiency disorders [5,6]. If left untreated, thyroid disorders may lead to complications that may impact the patients' quality of life [7].

The clinical symptoms of a thyroid disorder mainly depend on the type of the disorder and may affect different systems of the body. Furthermore, since most of the symptoms are not specific, thyroid disorders can be easily missed or confused with other medical conditions [2].

Thyroid disorders are one the most under-diagnosed and neglected medical conditions, and the lack of general knowledge among patients may be of considerable concern [8]. A previously done study in 2019, the central region of Saudi Arabia (KSA), shows a general lack of knowledge in the region despite the increasing prevalence of the condition [1]. Knowledge of thyroid disorder can help many people who have thyroid dysfunction, and they are not aware of their problem [9]. In general, lack of knowledge and comprehension of the thyroid gland and its symptoms will lead patients to go undiagnosed [8].

There is a limited number of studies in Saudi Arabia that assess knowledge and correlation to the diagnosis of thyroid disorders among the general population, particularly on the regional level. Moreover, no study was performed in the Eastern Province about this problem. Hence, this study aims to figure out general knowledge about thyroid disorders manifestations, risk factors, and preventive behaviors among the Eastern Province population of Saudi Arabia.

## Materials and methods

This cross-sectional study was conducted among 882 participants in the Eastern province of Saudi Arabia from July 2020 to October 2020, were selected randomly. The representative sample included Saudi and non-Saudi males and females of different age groups (18-60 years). A self-administrated online questionnaire translated into Arabic was used to gather information on the following variables: socio-demographics, knowledge of the thyroid gland, its function, its disorders, risk factors affecting thyroid disorders, and past medical history of thyroid disorders. The questionnaire was subjected and tested using the Cronbach alpha test to ensure its reliability and validity.

The criteria for the knowledge regarding thyroid disease was drawn from 17 questions where "yes" coded as 1 "no/I don't know" coded as 0 were the answer options. The total knowledge score has been calculated by adding all questions. A possible score range from 0 to 17 has been generated, which indicates that the higher the score, the higher the knowledge regarding thyroid disease, and by using 50% and 75% of the total score, participants were classified as having low knowledge if the score range was from 0 to 8 points, 9 to 12 points were classified as average knowledge, and 13 to 17 points were considered as high knowledge.

Descriptive statistics are presented using numbers, percentages, median (min-max), mean and standard deviation, whenever appropriate. Statistical collinearity was measured using Shapiro Wilk, Kolmogorov, and Smirnov tests. Between comparisons, Mann Whitney U test and Kruskal Wallis test were applied (non-parametric tests). All statistical analyses were carried out using Statistical Packages for Software Sciences (SPSS) version 21 (IBM Corporation, Armonk, New York). A P-value of <0.05 was considered statistically significant. Participants were informed that participation was entirely voluntary. Informed consent was provided in the survey for each participant, no identities were documented on the questionnaires, and all the participants' personal information was kept confidential. The study was approved by the Ethics Research Committee at the College of Medicine, King Faisal University.

## Results

This cross-sectional study recruited 882 participants to measure the knowledge regarding thyroid disease manifestations, its risk factors, and preventive behaviors. More than a half (54.2%) were in the younger age group (18-35 years) and females were more (55.7%). The prevalence of respondents with previous history and family history of thyroid diseases were 17.1% and 40.7%, respectively, and the most commonly mentioned thyroid diseases being detected was hypothyroidism.

The most common risk factors of thyroid diseases were insufficient or excess iodine intake (61.3%), followed by pregnancy and the postpartum period (61.1%) and radiation exposure (57.4%), while the most frequently mentioned symptoms were fatigue (81.7%), followed by neck lump (70.6%) and feeling cold and weight gain (68.9%). The overall mean knowledge score was 8.67 (SD 3.69), with 44.7%, 41.2%, and 14.2% were classified into low, average, and high knowledge, respectively.

By comparing the mean knowledge score among the socio-demographic profiles and previous history of thyroid disease, the current study shows that being a female, living in Al Ahsa, being a student, those with a previous history of thyroid disease, family history of the disease, and those who underwent thyroid gland examination were significantly more associated with having better knowledge toward thyroid diseases.

Table 1 presents the socio-demographic characteristics of the 882 respondents. The most common age group was 18-35 years (54.2%), with more than half were females (55.7%), and nearly all were Saudis (97.6%). Regarding residence location, half of them (50.7%) were living in Dammam and Al Khobar, and 41.5% were living in Al Ahsa. Concerning educational attainment, nearly all (81.5%) were professionals, and more than one-third (36.4%) were currently employed. In addition, 68% earned less than 120,000 SAR per year.

**Table 1:** Socio-demographic characteristics of participants (n=882)

Study variables	N (%)
Age group	
18–35 years	478 (54.2%)
36–50 years	204 (23.1%)
>50 years	200 (22.7%)
Gender	
Male	391 (44.3%)
Female	491 (55.7%)
Nationality	
Saudi	861 (97.6%)
Non-Saudi	21 (02.4%)
Residence area	
Al Ahsa	366 (41.5%)
Dammam and Alkhobar	447 (50.7%)
Others	69 (07.8%)
Marital status	
Unmarried	405 (45.9%)
Married	477 (54.1%)
Educational level	
High school or below	163 (18.5%)
Bachelor or higher	719 (81.5%)
Employment	
Employed	321 (36.4%)
Unemployed	318 (36.1%)
Student	243 (27.6%)
Annual income (SAR)	
<120,000	600 (68.0%)
≥120,000	282 (32.0%)

Table 2 describes the past medical history of the respondents. The proportion of respondents who had a previous history of thyroid disease was 17.1%, and hypothyroidism was the most commonly detected disease (68.9%). Furthermore, a family history of thyroid disease was also reported by 40.7% of the respondents, with hypothyroidism was also the most commonly mentioned disease (59.1%). The proportion of respondents who underwent thyroid gland examinations were 37.9%, and the most frequently mentioned reason was for a routine checkup (39.2%).

**Table 2:** Past medical history of thyroid diseases (n=882) n: responders who answer this question.

Statement	N (%)
Previous diagnosis with thyroid disease	
Yes	151 (17.1%)
No	731 (82.9%)
Type of thyroid diseases^(n=151)^	
Hypothyroidism	104 (68.9%)
Hyperthyroidism	15 (09.9%)
Thyroid nodule	17 (11.3%)
Thyroid cancer	01 (0.70%)
I don’t know	14 (09.3%)
Family history of thyroid disease	
Yes	359 (40.7%)
No	523 (59.3%)
Type of thyroid disease diagnosed with relatives^(n=359)^	
Hypothyroidism	212 (59.1%)
Hyperthyroidism	45 (12.5%)
Thyroid nodule	22 (06.1%)
Thyroid cancer	13 (03.6%)
I don’t know	67 (18.7%)
Underwent thyroid gland investigations	
Yes	334 (37.9%)
No	548 (62.1%)
Reason for thyroid gland investigations^(n=334)^	
Routine check	131 (39.2%)
Doctor’s suggestion	76 (22.8%)
Symptoms of thyroid	92 (27.5%)
Neck swelling	19 (05.7%)
I don’t know	16 (04.8%)

Table 3 shows the assessment of knowledge regarding the risk factors, manifestations, and preventive behaviors. Based on the results, respondents showed adequate knowledge for the following risk factors including; insufficient or excess iodine intake (61.3%) followed by pregnancy and the postpartum period (61.1%) and radiation exposure (57.4%) while it was least on taking Amiodarone medication (7.8%). For the knowledge about the clinical manifestation of thyroid diseases, respondents were knowledgeable for the following symptoms of thyroid diseases that include; fatigue (81.7%), followed by neck lump (70.9%) and feeling cold and weight gain (68.9%) while diarrhea, constipation or stomach ache were the least knowledge symptoms of thyroid diseases (28.2%). Regarding the knowledge about preventive behavior, respondents exhibited good knowledge about the early thyroid function tests to prevent the complication of thyroid diseases (90.2%), while they showed poor knowledge regarding being away from Soy food as a preventive way of thyroid diseases among women (13.7%).

**Table 3:** Assessment of knowledge regarding risk factors, manifestations, and preventive behaviors of participants (n=882)

Statement	Yes (%)
Knowledge of risk factors	
Do you think smoking is a risk factor for thyroid diseases?	357 (40.5%)
Do you think radiation exposure is a risk factor for thyroid diseases?	506 (57.4%)
Do you think insufficient or excess iodine intake is a risk factor for thyroid diseases?	541 (61.3%)
Do you think females are more at risk of having thyroid diseases?	273 (31.0%)
Do you think the pregnancy and the postpartum period are risk factors for thyroid diseases?	539 (61.1%)
Do you think the medication Amiodarone (known commercially as Pacerone, Cordarone, Advadarone, Sedacoron) is a risk factor for thyroid diseases?	69 (07.8%)
Do you think lithium intake is a risk factor for thyroid diseases?	121 (13.7%)
Knowledge about the clinical picture of thyroid disorders	
Do you think feeling cold and weight gain are common symptoms of having hypothyroidism?	608 (68.9%)
Do you think feeling hot and weight loss are common symptoms of having hyperthyroidism?	559 (63.4%)
Do you think the neck lump can be a sign of thyroid diseases?	623 (70.6%)
Do you think fatigue can be a symptom of thyroid diseases?	721 (81.7%)
Do you think diarrhea, constipation, or stomachache can be symptoms of thyroid diseases?	249 (28.2%)
Do you think skin and nail changes or hair loss can be signs of thyroid diseases?	378 (42.9%)
Do you think bulging eyes can be a sign of thyroid diseases?	505 (57.3%)
Knowledge about the prevention of thyroid disorders	
Do you think being away from Soy food is one of the preventive way from thyroid diseases in women?	121 (13.7%)
Do you think early thyroid function tests can prevent the complication of thyroid diseases?	796 (90.2%)
Do you think a well-balanced diet is essential to prevent thyroid diseases?	681 (77.2%)

Table 4 presents the descriptive statistics of the knowledge regarding thyroid disease. The mean score of the knowledge regarding risk factors, manifestation, and preventive behavior was 2.73, 4.13, and 1.81, with mean percentages of 39%, 59%, and 60.3%, respectively. The overall mean knowledge score was 8.67 (SD 3.69), with a mean percentage of 51%.

**Table 4:** Descriptive statistics of the overall knowledge regarding thyroid disease (n=882)

Variables	No. of items	Mean ± SD	Mean (%)	Median (min–max)
Knowledge regarding risk factors	07	2.73 ± 1.63	39.0	3.00 (0–7.00)
Knowledge regarding manifestation	07	4.13 ± 2.14	59.0	4.00 (0–7.00)
Knowledge regarding preventive behavior	03	1.81 ± 0.75	60.3	2.00 (0–3.00)
Total knowledge score	17	8.67 ± 3.69	51.0	9.00 (0–17.00)

**Figure 1:**
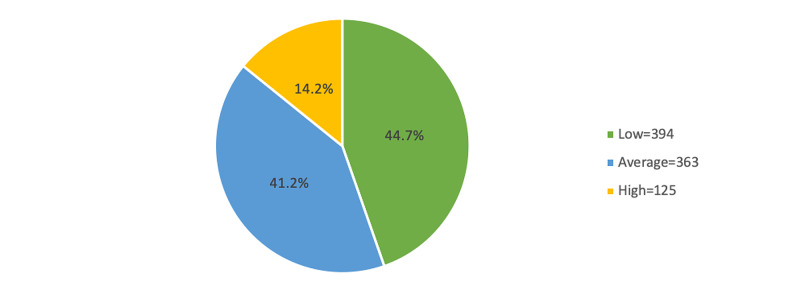
Level of knowledge regarding thyroid disease

Figure 1 depicts the level of knowledge regarding thyroid diseases. Based on the given criteria, it was revealed that approximately 45% were classified as low knowledge, 41.2% were classified as average knowledge, and the rest (14.2%) were classified as high knowledge.

Table 5 presents measuring the differences in knowledge in relation to the socio-demographic characteristics and the previous history of thyroid diseases. It was found that the knowledge of respondents was statistically significantly higher in the following variables, including; being a female (T=−6.920; p<0.001), living in Al Ahsa (F=11.368; p<0.001), being a student (F=6.931; p=0.003), previous history of thyroid disease (T=3.384; p<0.001), family history of thyroid disease (T=5.076; p<0.001), and previous history of thyroid gland examination (T=5.771; p<0.001) while age group, nationality, marital status, educational level, and annual income did not show a significant difference when compared to the overall knowledge score (all p>0.05).

**Table 5:** Statistical differences between the mean knowledge score among the participants' socio-demographic characteristics and the previous history of thyroid disease (n=882) ^a^P-value has been calculated using the Kruskal Wallis test. ^b^P-value has been calculated using the Mann Whitney U test. **Significant at p<0.05 level.

Factor	Knowledge score (17) mean ± SD	T/F-test	P-value
Age group^a^			
18–35 years	8.56 ± 4.08	F=1.675	0.134
36–50 years	9.08 ± 3.05		
>50 years	8.52 ± 3.25		
Gender^b^			
Male	7.73 ± 4.33	T=−6.920	<0.001**
Female	9.42 ± 2.88		
Nationality^b^			
Saudi	8.68 ± 3.65	T=0.663	0.629
Non-Saudi	8.14 ± 5.11		
Residence area^a^			
Al Ahsa	9.33 ± 3.69	F=11.368	<0.001**
Dammam and Alkhobar	8.11 ± 3.64		
Others	8.78 ± 3.44		
Marital status^b^			
Unmarried	8.59 ± 4.17	T=−0.556	0.258
Married	8.73 ± 3.23		
Educational level^b^			
High school or below	8.75 ± 3.54	T=0.324	0.749
Bachelor or higher	8.65 ± 3.72		
Employment status^a^			
Employed	8.17 ± 3.69	F=6.931	0.003**
Unemployed	8.68 ± 3.16		
Student	9.33 ± 4.21		
Annual income (SAR)^b^			
<120,000	8.67 ± 3.73	T=−0.040	0.927
≥120,000	8.68 ± 3.60		
Previous diagnosis with thyroid disease^b^			
Yes	9.59 ± 2.87	T=3.384	<0.001**
No	8.48 ± 3.81		
Family history of thyroid disease^b^			
Yes	9.42 ± 3.17	T=5.076	<0.001**
No	8.15 ± 3.93		
Underwent thyroid gland investigations^b^			
Yes	9.57 ± 2.84	T=5.771	<0.001**
No	8.12 ± 4.03		

## Discussion

Thyroid disorders are one of the most underdiagnosed and neglected medical problems, and the lack of general patient knowledge may be of considerable concern [8]. Awareness can benefit many individuals who have thyroid disease and are unaware of their problem [9].

This cross-sectional community-based study was conducted to evaluate the awareness of the thyroid gland, its function, its disorders, risk factors affecting thyroid disorders, and past medical history of thyroid disorders among the general population of the eastern province of Saudi Arabia. The results of this study provide unique information on patterns of eastern province population-level of awareness, knowledge, and past medical history of thyroid disorder.

In the current study, the total number of participants was 882, where the overall mean knowledge score was 8.67, were (44.7%) classified into low knowledge, (41.2%) average, and (14.2%) high. Comparing the mean knowledge score between the socio-demographic profiles and the previous history of thyroid disease, being female, living in Al Ahsa, students, those with a previous history of thyroid disease, family history of the disease, and those undergoing thyroid tests, was significantly more associated with better knowledge of thyroid disease.

Poor knowledge of thyroid disorders is an issue in many countries [3]. According to a study done in India that shows similar results to this study, most of the participants had inadequate knowledge and misconceptions of the thyroid gland and associated disorders [8]. On the contrary, a study that was done in Riyadh (capital of Saudi Arabia) shows a different level of knowledge where 57% of the participant had a good level of knowledge [10]. Furthermore, the current study shows that there is a significant difference between genders. Females are significantly more knowledgeable about thyroid disease. Compared with a study conducted in Saudi Arabia, there is no significant difference between gender in the level of knowledge [10].

Awareness of clinical features among this study population provides some interesting points of agreement and disagreement with other studies. For instance, the current study population answered that hypothyroidism is associated with weight gain (68.9%) and 63.4% of the responders believe that weight loss changes are associated with hyperthyroidism. In comparison, the study by Rai et al., as around (50%) answered that weight gain is characteristic of hypothyroidism. Moreover, the study by Rai et al. showed that it is about 61% think fatigue is a symptom, while the current study concludes 81.7% agreeing with this information [8]. On the other hand, a study conducted in Saudi Arabia suggested that 25.3% of the population responded by recognizing the relationship of neck swelling, constipation, and diarrhea as symptoms of thyroid disease. But the current study shows that 70.6% know about neck swelling and 28.2% about constipation and diarrhea, which conclude the latter one agreed with the previous study [2].

Knowledge about risk factors related to thyroid disorders is an important aspect. As per Wiersinga, smoking is considered a risk factor for thyroid diseases in many different physiological and pathological patterns. Considering these facts, this study shows that 40.5% of responders agreed that smoking is a risk factor for thyroid diseases [11]. Other parameter shows that being a female is a risk factor for thyroid disease. The current study shows that only 31% of the responders agree that being female increases the risks of having thyroid disease. On the other hand, a study conducted in Saudi Arabia by Aladwani et al. states that 21% strongly agree that female sex is a risk factor. So, those studies agreed that the majority of the population has a lack of knowledge on this point, which may delay the diagnosis [12].

Amiodarone is known as a risk factor for induced thyroid dysfunction. This has been confirmed as the study by Takeuchi et al. shows that around 30% prevalence of thyroid dysfunction among patients on amiodarone therapy. The current study shows a significant lack of knowledge about 8% only respond that amiodarone is a risk factor for thyroid disease [13]. A study conducted on patients with mood disorders who are using lithium shows an increase in thyroid-stimulating hormone (TSH) and thyroid volume. This has been seen as only 13.7% of the population know that lithium is considered a risk factor [14].

A few ways can be done to eliminate the possibility of developing spontaneous autoimmune thyroid dysfunction; however, early recognition can prevent overt disease progression. It is suggested that certain groups are more likely to benefit from screening programs, including those with atrial fibrillation, type 1 diabetes, hyperlipidemia, breast cancer, down syndrome, and Klinefelter syndrome [15]. In the current study, participants who were involved in any means of screening for thyroid disorders had significantly better knowledge regarding those conditions than others. This emphasizes that screening for thyroid dysfunction will help prevent complications from developing and improving thyroid disorders awareness.

Table 2 describes the past medical and past family history of thyroid disorders among the participants. Of all the respondents, 17% had a history of thyroid disorders, and 40% with a family history of thyroid disorders, with hypothyroidism being the most common in both groups. The past medical and past family history were both significantly correlated with better knowledge regarding thyroid disorders. This is consistent with what Canaris et al. have described in their article, which demonstrated that a higher prevalence of thyroid disorders is correlated with an increase in the level of knowledge about the manifestation of these conditions as well as the increased willingness for participating in thyroid screening initiatives [9].

The majority of participants agreed that following a well-balanced diet and screening for thyroid disorders can prevent and possibly decrease the chance of developing complications. However, most of the participants were not aware that avoiding food containing a high amount of soy can be considered a preventive measure against the development of thyroid disorders. These results are consistent with what Alotaibi and coworkers have described in their article [1].

## Conclusions

Nearly, half of the studied sample has low awareness scores regarding thyroid disease manifestations and its risk factors. The health authorities should hold more successful health education methods to improve the public and their caregivers' awareness of the various aspects of thyroid disorders and the value of their early detection and adequate control. Increased awareness and knowledge of their thyroid condition would enable patients to become more drug-compliant, follow-up on a regular basis, and distribute the right information to their family and friends.
